# M1-like macrophages change tumor blood vessels and microenvironment in murine melanoma

**DOI:** 10.1371/journal.pone.0191012

**Published:** 2018-01-10

**Authors:** Magdalena Jarosz-Biej, Natalia Kamińska, Sybilla Matuszczak, Tomasz Cichoń, Jolanta Pamuła-Piłat, Justyna Czapla, Ryszard Smolarczyk, Daria Skwarzyńska, Klaudia Kulik, Stanisław Szala

**Affiliations:** Center for Translational Research and Molecular Biology of Cancer, Maria Sklodowska-Curie Memorial Cancer Center and Institute of Oncology, Gliwice Branch, Gliwice, Poland; Mie University Graduate School of Medicine, JAPAN

## Abstract

Tumor-associated macrophages (TAMs) play a significant role in at least two key processes underlying neoplastic progression: angiogenesis and immune surveillance. TAMs phenotypic changes play important role in tumor vessel abnormalization/ normalization. M2-like TAMs stimulate immunosuppression and formation of defective tumor blood vessels leading to tumor progression. In contrast M1-like TAMs trigger immune response and normalize irregular tumor vascular network which should sensitize cancer cells to chemo- and radiotherapy and lead to tumor growth regression. Here, we demonstrated that combination of endoglin-based DNA vaccine with interleukin 12 repolarizes TAMs from tumor growth-promoting M2-like phenotype to tumor growth-inhibiting M1-like phenotype. Combined therapy enhances tumor infiltration by CD4^+^, CD8^+^ lymphocytes and NK cells. Depletion of TAMs as well as CD8^+^ lymphocytes and NK cells, but not CD4^+^ lymphocytes, reduces the effect of combined therapy. Furthermore, combined therapy improves tumor vessel maturation, perfusion and reduces hypoxia. It caused that suboptimal doses of doxorubicin reduced the growth of tumors in mice treated with combined therapy. To summarize, combination of antiangiogenic drug and immunostimulatory agent repolarizes TAMs phenotype from M2-like (pro-tumor) into M1-like (anti-tumor) which affects the structure of tumor blood vessels, improves the effect of chemotherapy and leads to tumor growth regression.

## Introduction

Progression of tumor strongly depends on the tumor microenvironment [1–7]. Cells that form tumor milieu are cells of mesenchymal origin (among others: fibroblasts, myofibroblasts, mesenchymal stromal cells (MSC)); immune cells (among others: monocytes, macrophages, neutrophils, T and B lymphocytes, dendritic cells, immunosuppressive T_reg_ cells, myeloid-derived suppressor cells (MDSC) and cells of the vascular system (including endothelial cells and pericytes) [2,7]. Normal cells found in tumors participate in immunosuppression and formation of tumor vascular system. It is so because normal cells release proangiogenic agents which also act as immunosuppression stimulants [1,7–10]. The process of tumor blood vascular network development considerably affects growth and progression of cancer cells [11–15]. Structure of tumor blood vessels is defective and they are functionally abnormal [6,15–19]. Slowed-down circulation of blood leads to underoxygenation (hypoxia) and necrosis of cells present in the vicinity of the vessels [6,20].

A particular tropism to underoxygenated tumor regions has been demonstrated for macrophages which may represent ca. 50% of tumor mass [21,22]. Hypoxia results in phenotype reprogramming of macrophages [23–27]. From proinflammatory, antigen-presenting cells (the so-called M1 phenotype) these macrophages become anti-inflammatory. They also lose their ability to present antigens and start releasing proangiogenic and immunosuppressive factors (leading to M2 phenotype) [25,28]. M2-like macrophages induce T_reg_ lymphocytes and also other types of T-cell responses without antitumor activity. Whereas M1-like macrophages stimulate naïve T cells to elicit a Th1/ cytotoxic response [29]. So, M1-like cells can inhibit tumor growth whereas M2-like cells stimulate it [24,30–34]. While M2-like cells participate in the formation of abnormal dysfunctional blood vessels, M1-like cells tend to “normalize” tumor blood vasculature [35–38]. M1 cells release, among others, IL-12, TNF-α and iNOS, whereas cells displaying M2 phenotype produce IL-10 and TGF-β. Besides hypoxia, M1→M2 polarization is triggered by certain growth factors (such as VEGF, PlGF and GM-CSF), cytokines (such as IL-4, IL-6, IL-10 and IL-13) as well as chemokines (such as CCL22) [24,37,39]. Polarization is an important element of tumor progression: it contributes to proangiogenic and immunosuppressive tumor microenvironment [5,25,32].

Combination of antiangiogenic drug and immunostimulatory agent should revert TAMs phenotype from M2-like towards M1-like. Repolarization of TAMs can normalize irregular tumor vascular network which should sensitize cancer cells to chemo- and radiotherapy and lead to tumor growth regression [35–37]. Our group has conducted studies of tumor microenvironment polarization using combination of endoglin-based DNA vaccine (ENG vaccine) with interleukin 12 (IL-12). In the strategy oral DNA vaccine directed against endoglin was used. This protein is overexpressed on the surface of activated vascular endothelial cells but also on some cancer cells (among others B16-F10) [40–44]. Endoglin plays important role in vascular remodeling [45] and blood vessel maturation during angiogenesis [46]. ENG-based DNA vaccine inhibits angiogenesis [42]. IL-12 gene therapy, in turn, acts as immunostimulant [47–50]. Combination of these two agents inhibited the growth of experimental B16-F10 murine melanoma tumors. High efficacy of this combination (30% of completely cured mice) is also likely due to the presence of endoglin on the surface of B16-F10 cells. Thus, ENG vaccine-stimulated immune response is directed against not only endothelial cells but cancer cells as well. We observed that combination of endoglin-based DNA vaccine with interleukin 12 reduced microvessel density and lowered the level of T_reg_ lymphocytes in tumors [42].

In this study, we examined repolarization of TAMs from M2- to M1-like phenotype in B16-F10 murine melanoma, exerted by a combination of endoglin-based DNA vaccine with IL-12 and the effect of this reversion on tumor blood vessels. Our results demonstrate that combination of ENG-based DNA vaccine with IL-12 significantly increases the percentage of the tumor-infiltrating M1-like macrophages (anti-tumor) and reduces percentage of the tumor-infiltrating M2-like macrophages (pro-tumor). Combined therapy enhances tumor infiltration by T lymphocytes and NK cells. Furthermore the structure of tumor vessels in mice treated with combined therapy resembles a regular one, which improves the antitumor effect of suboptimal doses of doxorubicin and leads to tumor growth regression.

## Materials and methods

### Bacterial strain, plasmids and cell line

The attenuated *Salmonella* Typhimurium SL7207 (aroA¯) strain was provided by Dr. C. A. Guzmán (German Research Center of Biotechnology, Braunschweig, Germany). Bacteria were cultured in LB broth supplemented with 100 μg/mL of ampicillin and with a mixture of amino acids. Endoglin-based DNA vaccine (*Salmonella* Typhimurium SL7207 strain carrying pcDNA3.1(+) plasmid with inserted endoglin (ENG) coding sequence) was used [42]. Plasmid pBCMGSNeo carrying a gene encoding murine IL-12 was obtained from Dr. H. Yamamoto (Osaka University, Osaka, Japan). Plasmid preparations were isolated using QIAGEN-Endo Free Giga Kit (QIAGEN GmbH, Hilden, Germany). B16-F10 cells (murine melanoma, ATCC, Manassas, VA, USA) were maintained using RPMI 1640 medium (Gibco BRL, Paisley, UK) supplemented with 10% FBS (ICN Biomedicals, Costa Mesa, CA, USA). Cell cultures were maintained under standard conditions (37°C, 5% CO_2_, 95% humidity) (see Jarosz et al. [42]).

### Mice and ethic statement

C57BL/6NCrl mice (six-to-eight-week old females, 18–22 g) were originally purchased from Charles River Laboratories (Wilmington, MA, USA). All mice were housed in the Maria Sklodowska-Curie Institute-Oncology Center, Gliwice Branch (Poland) in a HEPA-filtered Allentown’s IVC System (Allentown Caging Equipment Co, NJ, USA). Mice (n≤5) were kept in cages with an area of 435 cm^2^ and a height of 13,3 cm (Allentown Caging Equipment Co). The cage bed was a dust-free, resin-free, autoclavable litter of aspen wood (MAXI—LTE 004, ABEDD Vertriebs GmbH, Wien, Österreich). The environment was enriched with nesting materials of aspen wood fibers with 2.5 mm (NBF E-011, Allentown Caging Equipment Co). Mice were kept under 12-hour dark/12-hour light cycle in SPF animal facility. The relative humidity in the air-conditioned rooms was maintained at 50–55% and temperature at 21–22°C. The animals received a total pathogen-free standard diet (Altromin 1314, Altromin Spezialfutter GmbH & Co. KG, Germany) and water *ad libitum* throughout the whole study. This study was carried out in strict accordance with the recommendations in the Guide for the Care and Use of Laboratory Animals of the National Institutes of Health. The protocol was approved by the Committee on the Ethics of Animal Experiments of the Local Ethics Commission (Medical University of Silesia, Katowice, Poland) (Permit Number: 71/2013). All efforts were made to minimize animal suffering by qualified personal. After tumor cells injection, monitoring for animal health was performed every day (activity, appetite, behavior, and response to treatment). During this study only single animals from control group displayed symptoms of suffering or reached the termination criteria (weight loss > 20%, hunched posture, decreased activity/ locomotion) [51]. To alleviate the pain of these experimental animals euthanasia by cervical dislocation was conducted. Growing tumors were measured with calipers and tumor volumes were determined using the formula: volume = width^2^× length×0.52 [52]. Mouse were weighed during monitoring the size of the tumors. Mice whose tumor size exceeded 2 cm in any dimension (2.5 cm in individual cases) were sacrificed by cervical dislocation. During the experiments we observed no side effects of conducted therapy (the BCS was ≥ 4). Each procedure was terminated by cervical dislocation and tissue collection (tumors, spleen) for immunofluorescence analysis.

### Therapy

C57BL/6NCrl mice were injected subcutaneously on the left flank with 1×10^5^ B16-F10 cells in 100 μL PBS¯. One day after inoculating mice with B16-F10 cells, oral administrations of the ENG vaccine (1×10^8^ *cfu Salmonella* Typhimurium SL7207/ ENG per 100 μL PBS¯) were initiated. Bacteria were administered three times, one week apart. Five minutes before each administration, mice were given 100 μL 10% NaHCO_3_ in order to neutralize gastric acid. Additionally, on days 9, 11 and 13 following inoculation with cancer cells, pBCMGSNeo/mIL-12 plasmid (IL-12) was injected directly into tumors (20 μg DNA per 100 μL PBS¯) [42].

### Drug treatment and cell depletion

Doxorubicin (Sigma Aldrich, St Louis, MO, USA) was delivered intraperitoneally at a dose of 2.5 mg/kg, 3 times/week [34]. For TAMs depletion, liposomes (‘empty’ liposomes or Clodronate liposomes; Clodronate Liposomes Organisation, http://www.clodronateliposomes.org, Vrije Universiteit, Netherlands; [53]) were delivered intraperitoneally at a dose of 10 mg/kg and directly into tumors at a dose of 5 mg/kg, 2 times/week. For CD4^+^, CD8^+^ lymphocytes and NK cells depletion, monoclonal antibodies (anti-CD8a clone: 53.6.72 [36], anti-CD4 lymphocytes clone: GK1.5 [54] or anti-NK cells clone: PK136 [35]; BioXCell, West Lebanon, USA) were injected intraperitoneally at a dose of 200 μg per mouse on days -1 (1 day before the first vaccination), 1, 6, 11 and 16 to maintain the depletion status. Depletion was monitored on the 10^th^ day after B16-F10 inoculation by staining CD4^+^, CD8^+^ lymphocytes and NK cells in peripheral blood sample and in the end (on 20^th^ day) of experiment by staining CD4^+^, CD8^+^ lymphocytes and NK cells in spleens and tumors [36].

### Immunohistochemistry

For immunohistochemical analyses excited tumors were formalin-fixed and embedded in paraffin or frozen in OCT (CellPath, Newtown, UK) and liquid nitrogen. Tumors were sectioned into 5 μm slices. “Normalization” of blood vessels was assessed using several tests [35]. Paraffin sections were incubated with anti-α-Smooth Muscle Actin (αSMA) and anti-CD31 antibodies (Abcam, Cambridge, UK) and subsequently with Texas Red and FITC conjugated secondary antibodies (Vector Laboratories, Burlingame, CA, USA) respectively for pericytes coating vessels assessment; cancer cells that underwent apoptosis were stained by anti-caspase 3 antibody (Abcam, Cambridge, UK) and FITC-conjugated secondary antibody (Vector Laboratories); hypoxic regions in tumors were analyzed by Hypoxyprobe kit (Chemicon, Binerica, MA, USA) following the manufacturer’s instructions [55]. For assessment of blood vessels functionality in tumors, FITC-conjugated lectin (100 μg/100 μL, *Lycopersicion esculentum*, Vector Laboratories, Burlingame, CA, USA) was injected into mouse tail vein and allowed to circulate for 15 min prior 4% PFA perfusion. Subsequently tumors were excited, embedded in OCT and frozen in liquid nitrogen for later anti-CD31 staining [35]. Additionally, macrophages in tumor frozen section were stained with anti-F4/80 (AbD Serotec, Kidlington, UK) and anti-CD206 (Abcam) antibodies and Texas Red and FITC conjugated secondary antibodies (Vector Laboratories) respectively. Lymphocytes were stained with anti-CD8a (Bio-Rad, Hercules, CA, USA) antibody and Alexa Fluor 594 conjugated secondary antibody (Vector Laboratories). Tumor sections were counterstained with DAPI (Vector Laboratories). The results are expressed as percentage of area [%] calculated by ImageJ 1.48v where applicable.

### Flow cytometric analysis

Mice burdened with tumors were sacrificed by cervical dislocation. To macrophage isolation, single-cell suspensions were obtained using a collagenase II solution (500U/mL; Gibco BRL, Paisley, UK; [56]). Red blood cells were lysed using 0.15 M ammonium chloride solution (Sigma Aldrich). Cell suspensions were filtered using a 70-μm cell strainer. Dead cells were removed by centrifugation using Histopaque-1077 gradient (Sigma Aldrich). Cells were incubated with rat anti-mouse CD16/CD32 blocking antibody (eBioscience, San Diego, CA, USA) and then incubated with anti-CD11b, anti-F4/80 (eBioscience), anti-CD206 (Bio-Rad) antibodies for 30 min. All FACS-analyzed populations were gated in a DAPI¯ (Sigma Aldrich) window to enrich for live cells. To analyze macrophage phenotype [53], DAPI¯ viable tumor-derived cells and CD11b (to identify myeloid cells) were gated and then F4/80 (to identify TAMs) and CD206 (to identify M1-like) macrophages F4/80^+^/ CD206¯ and M2-like macrophages F4/80^+^/ CD206^+^). To identify the subpopulations of the myeloid cells (TAM, TAN and DC; [53]) in tumors, the following antibodies were used: anti-CD206, CD11b, F4/80 (eBioscience), Ly-6G, CD11c (BioLegend, San Diego, CA, USA). In flow cytometric analyses (BD FACSAria^™^ III; BD, Franklin Lakes, NJ, USA), gates dividing negative from positive cells were based on isotype antibody control probes. For T lymphocytes and NK cells isolation, single-cell suspension was obtained using a digestion mixture (0.5 mg/mL collagenase A, Sigma Aldrich; 0.2 mg/mL hyaluronidase type V, Sigma Aldrich; 0.02 mg/mL DNase I, Roche, Basel, Switzerland; per 0.25 g of tumor tissue). Red blood cells were lysed using 0.15 M ammonium chloride (Sigma Aldrich). Dead cells were removed by centrifugation using Lympholyte-M gradients (Cedarlane, Ontario, Canada). 7AAD (7-amino-actinomycin D; eBioscences) was used (10 μL/10^6^ cells) to stain nonviable cells just before running the flow analysis. To identify the subpopulations of T lymphocytes, 7AAD¯ viable tumor-derived cells were gated and then CD4 and CD8 (to identify T lymphocytes; BD Pharmingen, BD) or CD49b (to identify NK cells; eBioscences). In flow cytometric analyses (BD FACSCanto, BD), gates dividing negative from positive cells were based on isotype antibody control probes [52].

### Analysis of gene expression in macrophages at the mRNA level (Semi-quantitative real-time PCR)

RNA was isolated from DAPI¯/ CD11b^+^/ F4/80^+^ phenotype cells sorted using FACS AriaIII apparatus (BD). Total RNA samples were prepared using the Total RNA Mini Plus kit (A&A Biotechnology, Gdynia, Poland), according to manufacturer’s procedure. cDNA was synthesized with M-MuLV reverse transcriptase and random hexamers (EURx, Gdańsk, Poland). Starters characteristic for M1 and M2-specific cytokines and chemokines genes were employed (*mArg-1*, *mCCL-22*, *mMRC-1*, *mCCL-17*, *mCSF-1*, *mMMP-9*, *mIL-10*, *mCXCL-11*, *miNOS*, *mIL-1b*, *mCXCL-9*, *mIL-12a*, acc. to Huang et al. [36]; *mVEGFa*, *mPlGF* [57]; *mVEGFc* and *mNRP-1* [58]). mRNA levels’ measurements were performed by SG qPCR Master Mix (2x) (EURx, Gdańsk, Poland). The amplification was performed using a CFX96 Real-Time System (Bio-Rad, Hercules, CA, USA). Relative quantitation of mRNA was performed using ΔΔC_T_ method with β-actin as a reference gene. Means were calculated from three independent repeats.

### Statistical analysis

Tumor growth kinetics and statistical significance of differences between the experimental and control groups in immunological and immunohistochemical analyses were evaluated by difference tests. *P*-values<0.05 were considered statistically significant. Statistical analysis was performed using Statistica 10 software.

## Results

### Combination of ENG vaccine and IL-12 effectively inhibits tumor growth

**Fig 1:**
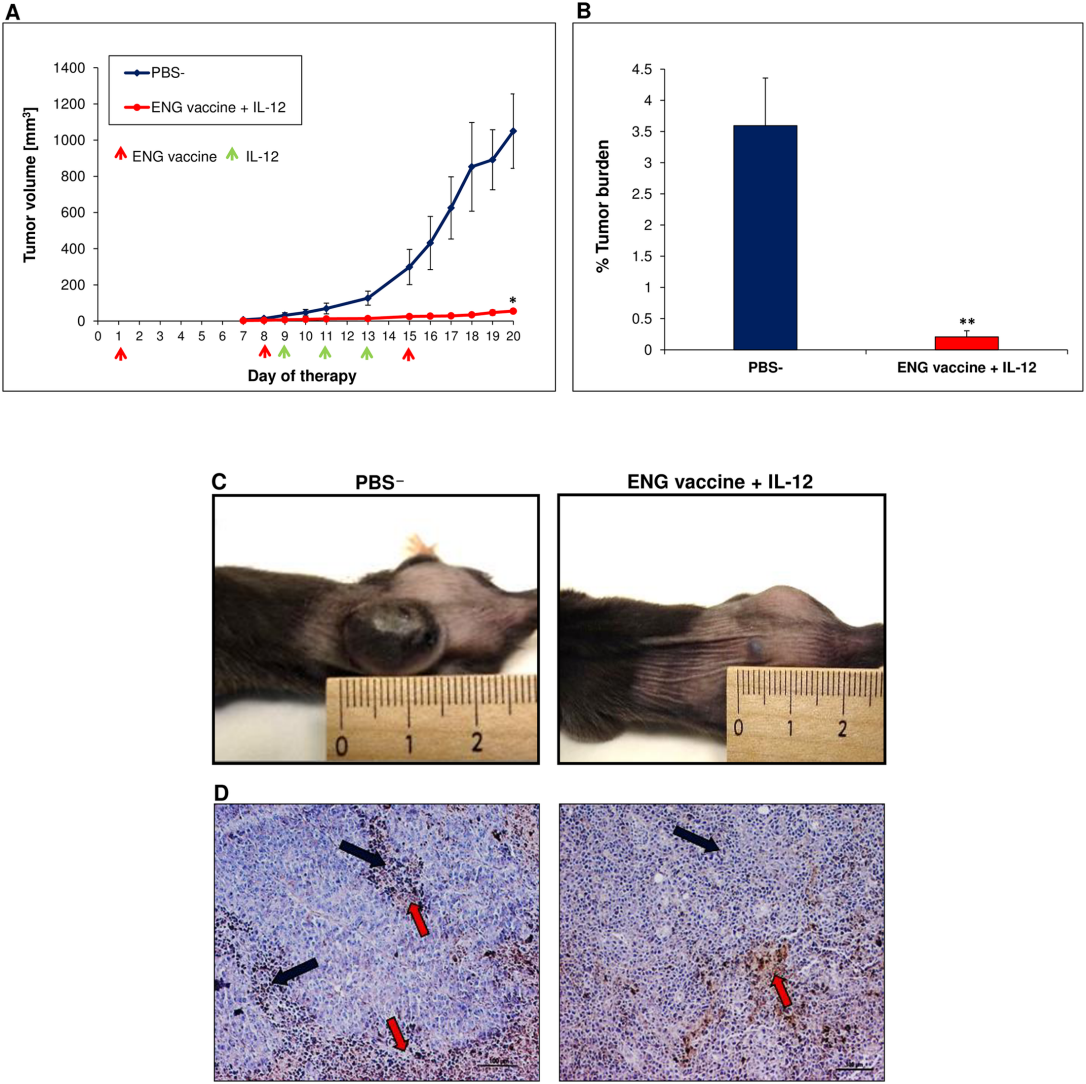
Inhibition of B16-F10 tumor growth in response to combined therapy involving endoglin-based DNA vaccine and IL-12. Mice (n = 10) were inoculated with B16-F10 cells (1x10^5^/animal) and then, on days 1, 8 and 15, SL7207/mCD105 bacteria were administered orally (1x10^8^ *cfu*/animal, in 100 μL PBS¯). Additionally, on days 9, 11 and 13 following inoculation with cancer cells, pBCMGSNeo/mIL-12 plasmid was injected directly into tumors (20μg DNA per 100 μL PBS¯). “Control” mice received PBS¯ only. Combined therapy was highly effective in inhibiting tumor growth compared to control. *P <0.001, the Cochran’s C test; **P <0.001, the Mann-Whitney U test. (A, B). Photographs were taken on 20^th^ day of therapy (C). Mice were sacrificed on 20^th^ day and tumor material was collected for H&E staining. Less necrotic areas (red arrows) and increased tumor infiltration by immune cells (black arrows) was observed in tumor sections from mice treated with combined therapy. Magnification 20× (D).

We examined the therapeutic effect of ENG vaccine and its combination with gene therapy mediated by plasmid DNA construct encoding murine interleukin-12. As we previously reported combined therapy is significantly more effective (as compared with monotherapies) in tumor growth inhibition [42]. Here, we observed more than 90% inhibition in tumor growth following ENG vaccine and IL-12 administration (see Fig 1). In the current study we have focused on investigation the role of macrophages in tumor regression following combined therapy.

### Endoglin-based DNA vaccine in combination with IL-12 changes the number and phenotype of tumor-infiltrating myeloid cells (from immunosuppressive to immunostimulatory)

We studied the effect of combined therapy on myeloid cells number and their phenotype. One day after inoculating mice with cancer cells the therapy was initiated with vaccine administration (on days 1, 8 and 15), followed by IL-12-mediated gene therapy (days 9, 11 and 13). Five days after last drug injection (on 20^th^ day) mice were sacrificed and tumors were collected. Subpopulations of myeloid cells were determined by immunohistochemistry and flow cytometry.

**Fig 2:**
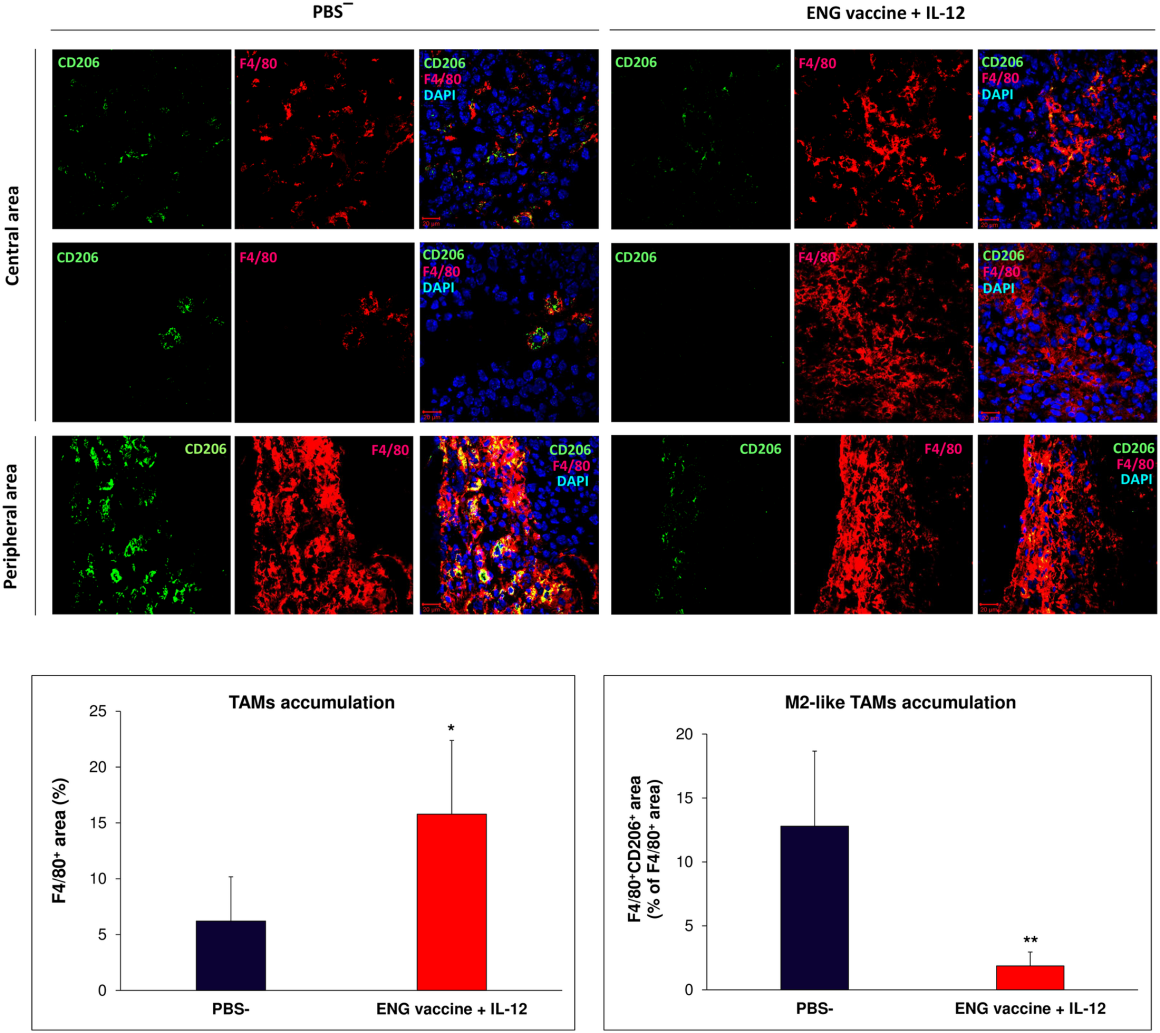
Effect of combined therapy (ENG vaccine + IL-12) on TAMs accumulation. One day after inoculating mice with B16-F10 melanoma cells, oral administration of endoglin-based DNA vaccine was initiated. ENG vaccine was administered three times, 1 week apart. Additionally, on days 9, 11 and 13 following inoculation with cancer cells, IL-12 was injected directly into tumors (see Materials and methods). On 20^th^ day of therapy, mice were sacrificed and tumors were excised for staining with antibodies against CD206 and F4/80. TAM macrophages (F4/80^+^) were located mainly on the peripheral zone in tumors of control mice and in central area of tumors of treated mice. Whereas M2-like macrophages (F4/80^+^/ CD206^+^) both in control and treated mice were present in necrotic areas of central zone and on the periphery of tumors. The area of TAM and M2-like TAM macrophages were determined from six tumors per group, in each tumor 8–12 visual field were analyzed (lens magnification: 20×). *P<0.0001, the Mann-Whitney *U* test.

In IHC sections of tumors from mice treated with combined therapy we observed a 2,5-fold increase of TAM macrophages (F4/80^+^) infiltration, as compared to control group. In control mice, macrophages were mainly located in peripheral areas of tumors, while in treated mice macrophages were also present in central area of tumor mass. In addition, in tumors of treated mice, we observed a 6-fold decrease in F4/80^+^/ CD206^+^ M2-like TAMs level. M2-like macrophages were located mainly on the periphery of the tumor and in central, necrotic areas both in tumors obtained from treated and control groups of mice (Fig 2).

**Fig 3:**
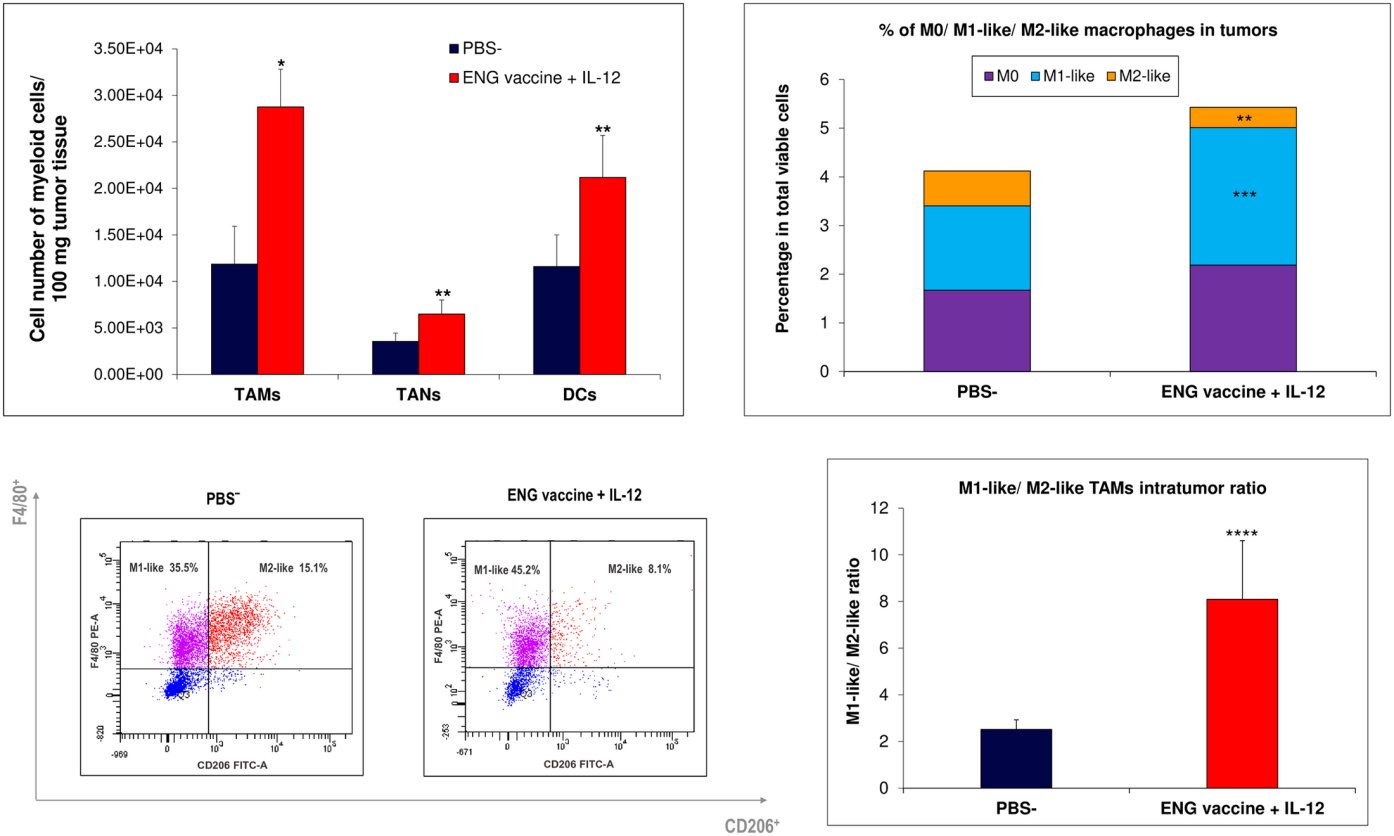
Effect of combined therapy (ENG vaccine + IL-12) on the myeloid cell phenotypes. One day after challenge with B16-F10 cells, oral administration of the ENG vaccine was initiated. ENG vaccine was administered three times, 1 week apart. Additionally, on days 9, 11 and 13 following inoculation with cancer cells, IL-12 was injected directly into tumors (Materials and methods). On the 20^th^ day of the experiment, tumors (n = 10) were excised. Obtained single-cell suspensions were then used to quantitate TAMs, TANs and DCs levels. Combined therapy increased macrophages (TAMs, defined as DAPI¯/ CD11b^+^/ CD11c¯/ Ly6-G¯), neutrophils (TANs, defined as DAPI‾/ CD11b^+^/ CD11c‾/ Ly-6G^+^) and dendritic cells (DCs, defined as DAPI¯/ CD11b^+^/ CD11c^+^/ Ly6-G¯) levels compared with control tumors. Furthermore, the ratio of M1-like (anti-tumor; defined as DAPI¯/ CD11b^+^/ F4/80^+^/ CD206¯) TAMs to M2-like (pro-tumor, defined as DAPI¯/ CD11b^+^/ F4/80^+^/ CD206^+^) TAMs was more than three times increased. *P<0.001, the Mann-Whitney *U* test; **P<0.02, the Student’s *t*-test; ***P<0.007, the Student’s *t*-test; ****P<0.007, the Cochran’s C test.

Cytofluorimetric analyses of tumors from mice treated with combined therapy have shown an increased (almost doubled) infiltration of TAMs (defined as CD11b^+^/ CD11c¯/ Ly6-G¯) as well as neutrophils (defined as CD11b^+^/ CD11c¯/ Ly-6G^+^) and DCs (defined as CD11b^+^/ CD11c^+^/ Ly6-G¯) as compared to control tumors. Moreover, within the population of tumor-infiltrating macrophages we observed increased subpopulation of M0 macrophages (defined as CD11b^+^/ F4/80¯/ Ly6-G¯) as well as M1-like macrophages (defined as CD11b^+^/ F4/80^+^/ CD206¯). Population of M2-like TAMs (defined as CD11b^+^/ F4/80^+^/ CD206^+^) was reduced over a half. The ratio of anti-tumor M1-like TAMs to pro-tumor M2-like TAMs was more than three times increased (Fig 3). This data demonstrates stimulation of cells of immune system by combined therapy and the shift of the phenotype of tumor-infiltrating macrophages towards the M1-like phenotype.

### Combined therapy changes gene expression of TAMs

**Fig 4:**
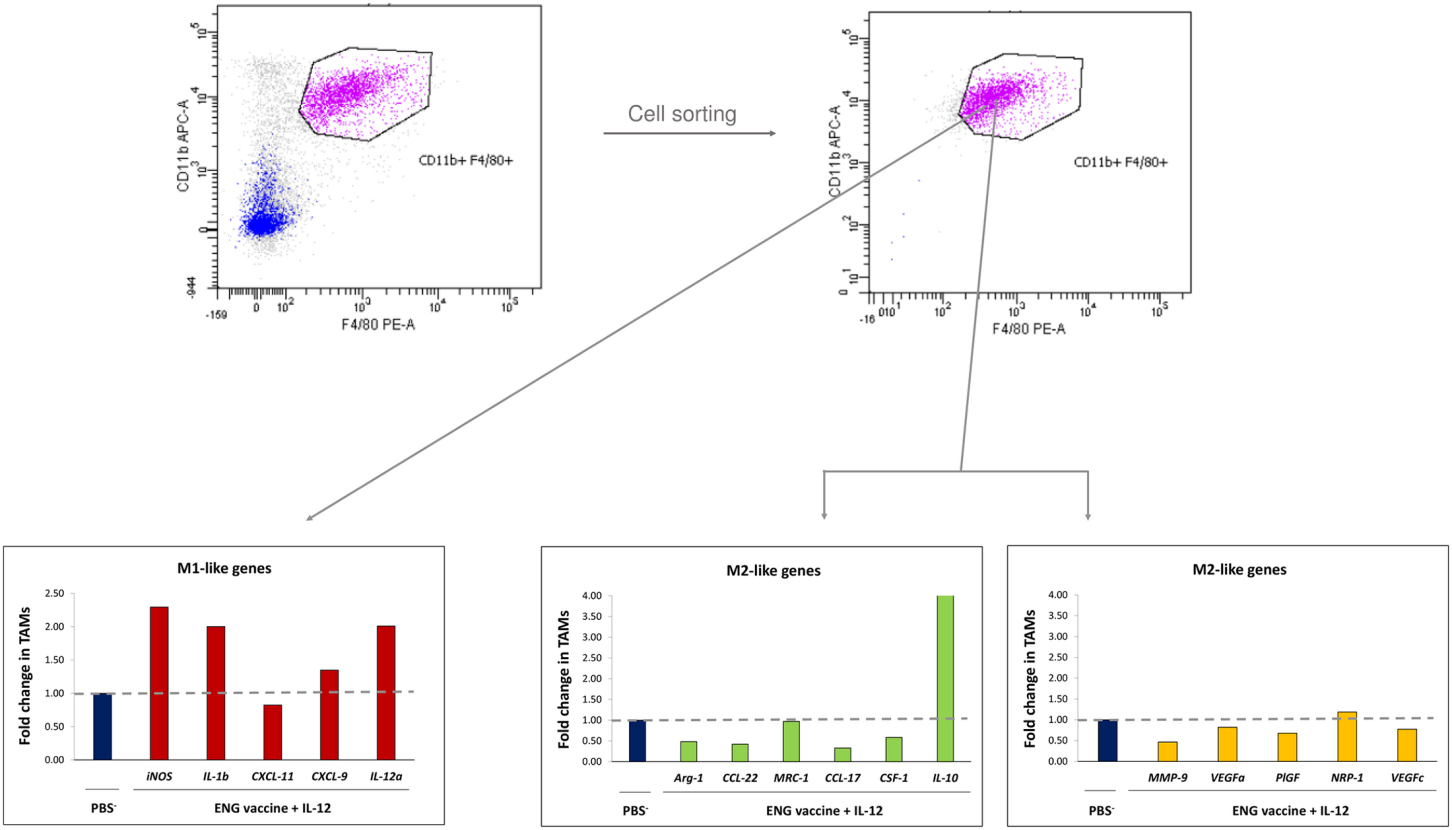
Effect of ENG vaccine in combination with IL-12 on cytokine expression in TAMs. On 20^th^ day of the combined therapy, mice were sacrificed and tumors were excised for TAMs FACS-sorting. TAMs (defined as DAPI¯/ CD11b^+^/ F4/80^+^) from 1–7 tumors were pooled as 4–5 samples in each group. Total RNA was extracted from sorted cells and converted to cDNA. Gene transcription in TAMs was analyzed by quantitative real-time PCR. Combined therapy up-regulated most of M1-like proinflammatory genes expression (red bars) and down-regulated most of M2-like anti-inflammatory (green bars) and proangiogenic (yellow bars) genes expression in TAMs as compared with controls. Horizontal dash: the value of 1 in controls.

We attempted to investigate the levels of M1-type and M2-type genes expression in tumor- infiltrating macrophages from mice treated with combined therapy (ENG vaccine + IL-12) and control mice (receiving PBS¯). For this purpose, on the 20^th^ day of therapy mice were sacrificed, and from collected tumors TAM macrophages were sorted (defined as CD11b^+^/ F4/80^+^). Using RT-PCR, we investigated the expression levels of genes specific for M2 and M1 macrophages. We observed increased expression of M1-type proinflammatory genes (*iNOS*, *IL-1b*, *CXCL-9*, *IL-12a*) and decreased expression of M2-type anti-inflammatory (*Arg-1*, *CCL-22*, *MRC-1*, *CCL-17*, *CSF-1)* and proangiogenic (*MMP-9*, *VEGFa*, *PlGF*, *VEGFc*) genes in TAMs from treated mice compared to controls. Only the level of *CXCL-11* gene expression, which is characteristic for M1 macrophages, was slightly reduced in tumors obtained from treated mice. In contrast, *IL-10* expression level, characteristic for M2 macrophages, was 4-times higher in tumors from treated mice compared to controls (Fig 4). In summary, combined therapy up-regulated most of M1-like gene expression and down-regulated most of M2-like gene expression in TAMs, as compared to controls.

### Depletion of TAMs abrogates antitumor effect of combined therapy

After revealing an induced repolarization towards M1-like cells following combined therapy we examined how important role is played by macrophages in inhibiting tumor growth by drug combinations. To investigate that, we used liposomes with clodronate to deplete macrophages *in vivo*. Liposomes (‘empty’ liposomes (control) or Clodronate liposomes (Clodr.)) were delivered intraperitoneally at a dose of 10 mg/kg and directly into tumor at a dose of 5 mg/kg, 2 times/week.

**Fig 5:**
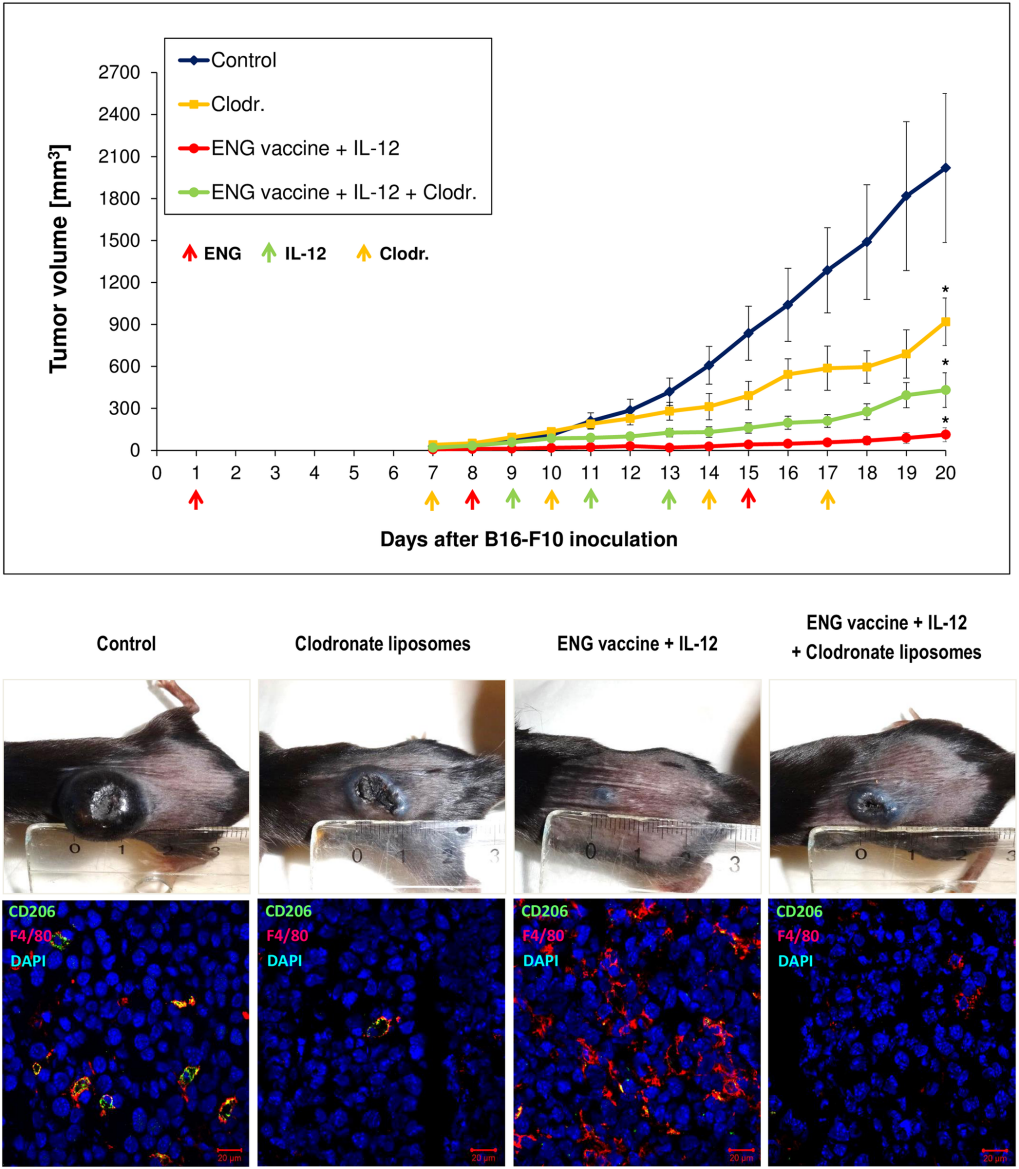
Depletion of macrophages reduces the effect of combining ENG-based DNA vaccine with IL-12. One day after inoculating mice (n = 7–8) with B16-F10 cells, the animals were orally vaccinated (three times at one-week intervals). Additionally, on days 9, 11 and 13 following inoculation with cancer cells, IL-12 was injected directly into tumors (Materials and methods). Moreover, liposomes (‘empty’ liposomes (control) or Clodronate liposomes (Clodr.)) were delivered intraperitoneally at a dose of 10 mg/kg and directly into tumors at a dose of 5 mg/kg, 2 times/week. Depletion of TAMs decreased the growth of control tumors. But in mice treated with combined therapy we observed enhanced tumor growth after TAMs depletion. Photographs were taken on 20^th^ day of the experiment. Tumors (n = 3) were collected 20 days after challenge and stained with antibodies against CD206 and F4/80. Magnification 20×. *P<0.0001, the ANOVA followed by the Tukey’s *post hoc* test.

We have observed that depletion of TAMs diminished the growth of control tumors. Furthermore in mice subjected to combined therapy we observed enhanced tumor growth after TAMs depletion. Comparing tumor-derived immunohistochemical specimens from mice treated with Clodronate liposomes and control (‘empty’ liposomes) we noted significant decrease in number of tumor-infiltrating macrophages in tumor sections in which TAMs depletion was achieved (Fig 5). These experiments indicate an important role of TAM macrophages in progression/regression of tumors.

### Endoglin-based DNA vaccine in combination with IL-12 enhances infiltration of T lymphocytes and NK cells into tumor

The next stage of the study was the investigation of the influence of TAMs phenotype changes on tumor infiltration by T-lymphocytes and NK cells. In our previous work, we observed increased levels of CD4^+^, CD8^+^ and NK cells after the use of the vaccine alone. Activated T lymphocytes were specifically targeted against endoglin expressing endothelial and tumor cells [42].

**Fig 6:**
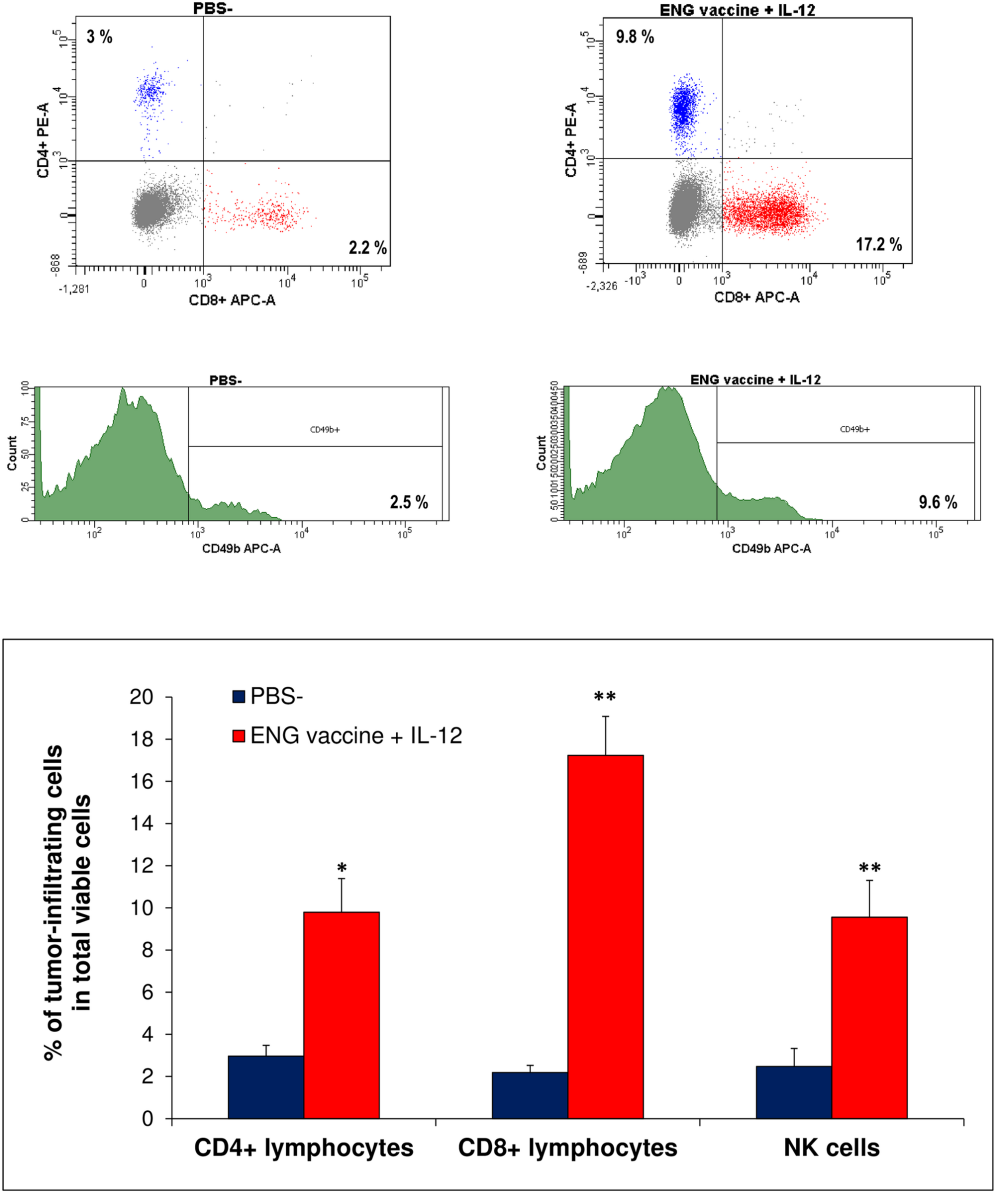
Effect of combined therapy (ENG vaccine + IL-12) on tumor-immune cells infiltration. On day 20^th^ of the combined therapy, mice (n = 9) were sacrificed and tumors were excised for flow analysis. Obtained single-cell suspensions were then used to quantitate the level of CD4^+^, CD8^+^ lymphocytes and NK cells. The percentage of CD4^+^, CD8^+^ lymphocytes and NK cells was determined in total viable cells (representative flow data for CD4^+^, CD8^+^ lymphocytes and NK cell populations). Combined therapy increased tumor-infiltrating CD4^+^ (more than three times), CD8^+^ lymphocytes (more than eight times) as well as NK cells (more than three times) levels compared with control tumors. *P<0.0001, the Cochran’s C test; **P<0.0001, the Student’s *t*-test.

On the 20^th^ day of the combined therapy, mice were sacrificed and tumors were excised for flow cytometric analyses. Single-cell suspensions were obtained and then used to quantitate CD4^+^, CD8^+^ lymphocytes and NK cells levels. We observed that combined therapy increased tumor-infiltrating CD4^+^ (more than three times), CD8^+^ lymphocytes (more than eight times) as well as NK cells (more than three times) levels compared with control tumors (Fig 6). This points the fact that changes in TAMs phenotype during combined therapy resulted in enhanced tumor infiltration by CD4^+^, CD8^+^ lymphocytes and NK cells.

**Fig 7:**
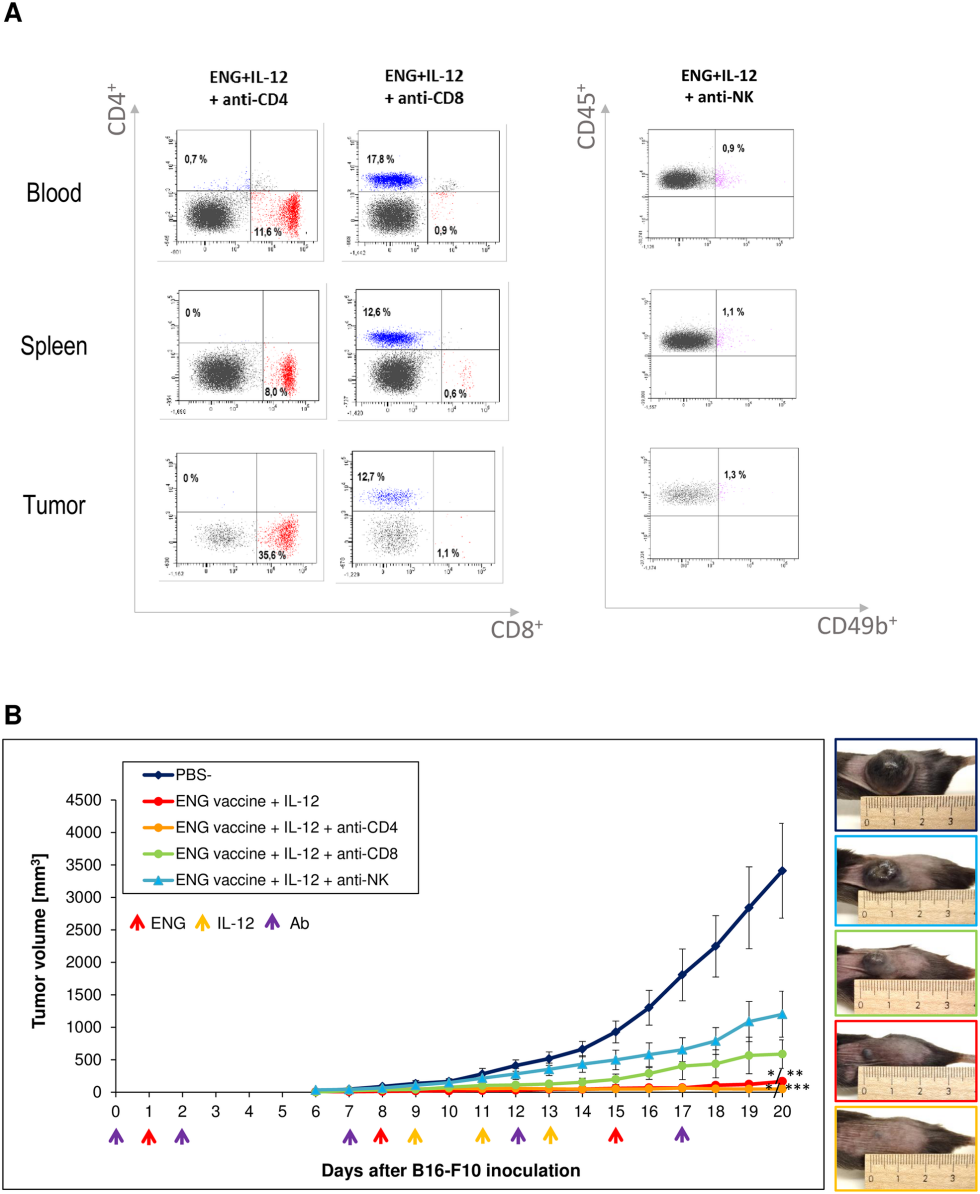
Depletion of CD8^+^ lymphocytes and NK cells, but not CD4^+^ lymphocytes, reduces the effect of combining ENG-based DNA vaccine with IL-12. One day after inoculating mice (n = 6–8) with B16-F10 cells, the animals were orally vaccinated (three times at one-week intervals). Additionally, on days 9, 11 and 13 following inoculation with cancer cells, IL-12 was injected directly into tumors (see Materials and methods). Moreover, monoclonal antibodies (anti-CD8a, anti-CD4 lymphocytes or anti-NK cells) were injected intraperitoneally at a dose of 200 μg per mouse on days -1 (1 day before the first vaccination), 1, 6, 11 and 16. Blood was collected on 10^th^ day after B16-F10 inoculation for flow analysis. Tumors and spleens were harvested 20 days after challenge and stained with antibodies against CD4, CD8 lymphocytes and NK cells. The percentage of CD4^+^, CD8^+^ lymphocytes and NK cells was determined in total viable lymphoid CD45^+^ cells. Representative flow data for CD4^+^, CD8^+^ lymphocytes and NK cell populations after antibody administration (A). Antibody selectively depleted cells in peripheral blood, spleens and tumors. Depletion of CD8^+^ lymphocytes and NK cells enhanced the growth of tumors in treated mice (more than 3 and 7 times, respectively). But after CD4^+^ lymphocytes depletion we observed more than 3 times decreased tumor growth in mice treated with combined therapy. Photographs were taken on 19^th^ day of the experiment (B). Magnification 20×. *P<0.0001 compared with control (PBS¯) group, **P<0.05 compared with NK depletion group, ***P<0.01 compared with NK depletion group; the Kruskal-Wallis followed by the *post hoc* multiple comparisons of rank sums test.

Next, we have examined which subpopulation of immune cells, as a result of combined therapy, plays a role in the inhibition of tumor growth. For this purpose we used antibodies for cell depletion (Fig 7A). Monoclonal antibodies (anti-CD8a, anti-CD4 lymphocytes or anti-NK cells) were injected intraperitoneally at a dose of 200 μg per mouse on days -1 (1 day before the first vaccination), 1, 6, 11 and 16. We observed that removal of CD8^+^ and NK cells abolishes the therapeutic effect of DNA vaccines in combination with IL-12. Tumor growth rate was increased more than three times after CD8^+^ depletion and more than seven times after NK cells depletion. In contrast, after CD4^+^ depletion, we observed more than three-fold reduction of the tumor mass in treated mice. Removal of CD4^+^ lymphocytes increased the therapeutic effect of endoglin-based DNA vaccine in combination with IL-12 (Fig 7B).

**Fig 8:**
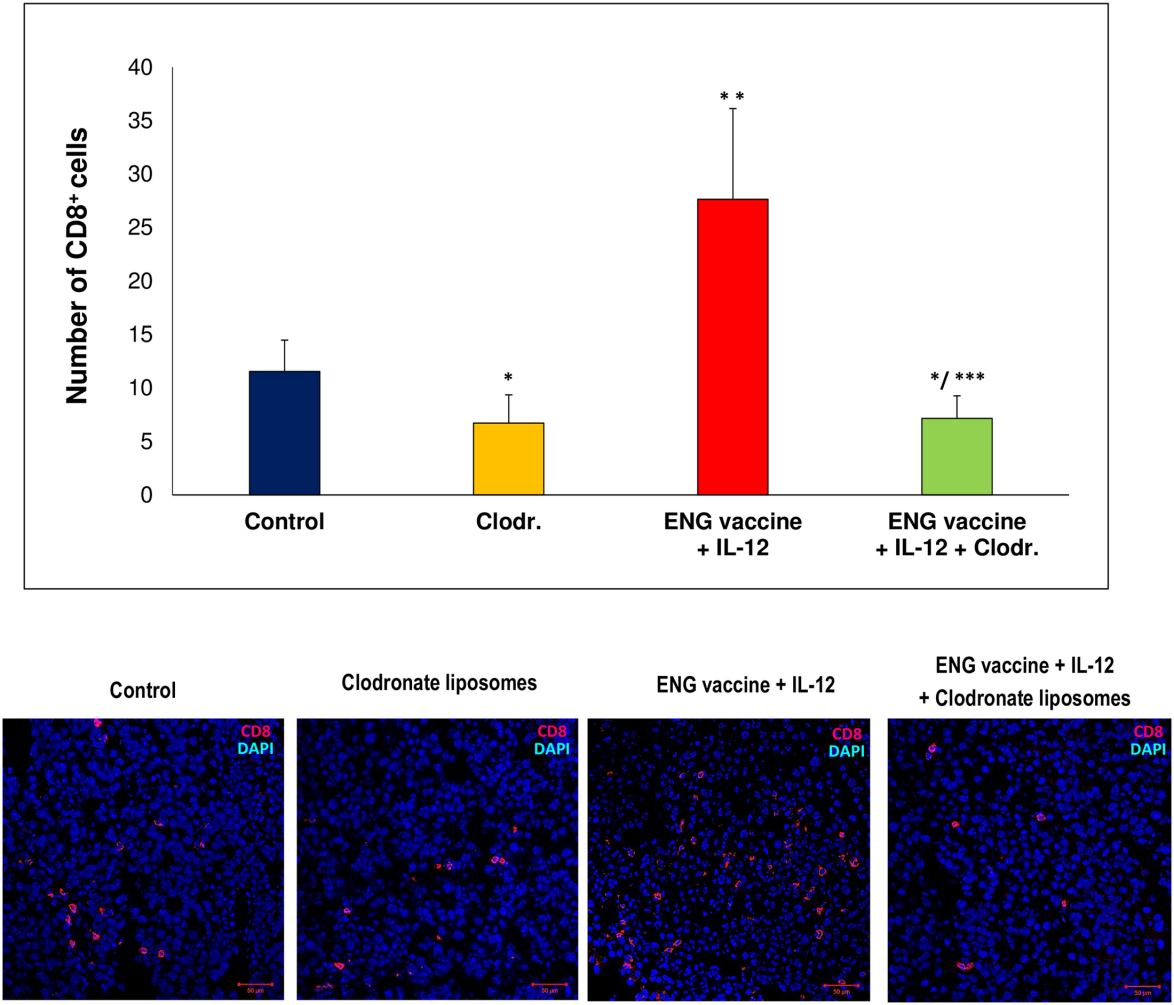
Depletion of TAMs changes accumulation of CD8^+^ T cells in tumors. One day after inoculating mice with B16-F10 cells, the animals were orally vaccinated (three times at one-week intervals). Additionally, on days 9, 11 and 13 following inoculation with cancer cells, IL-12 was injected directly into tumors. Moreover, liposomes (‘empty’ liposomes (control) or Clodronate liposomes (Clodr.)) were delivered intraperitoneally (see Materials and methods). Depletion of TAMs decreased recruitment of CD8^+^ T cells to control mice tumors, but in tumors of mice treated with combined therapy we observed over 75% decrease in CD8^+^ T cells infiltration. Tumors (n = 5) were collected 20 days after challenge and stained with antibody against CD8a. Magnification 20×. *P<0.005 compared with control (PBS¯) group, **P<0.00001 compared with control (PBS¯), Clodr. and ENG+IL-12 + Clodr. groups ***P<0.00001 compared with ENG+IL-12 group; the Kruskal-Wallis followed by the *post hoc* multiple comparisons of rank sums test.

In addition, we investigated whether depletion of macrophages affects the presence of T cells in tumors. The liposomes with clodronate was used to deplete macrophages *in vivo* (see Materials and methods). After the depletion of macrophages in tumors of control mice a reduced infiltration of CD8^+^ cells was observed. Whereas in treated mice more than 75% loss of CD8^+^ lymphocytes was observed (Fig 8). These experiments indicate an important role of TAM macrophages in the recruitment of CD8^+^ lymphocytes to tumors.

### Combined therapy changes tumor vasculature

**Fig 9:**
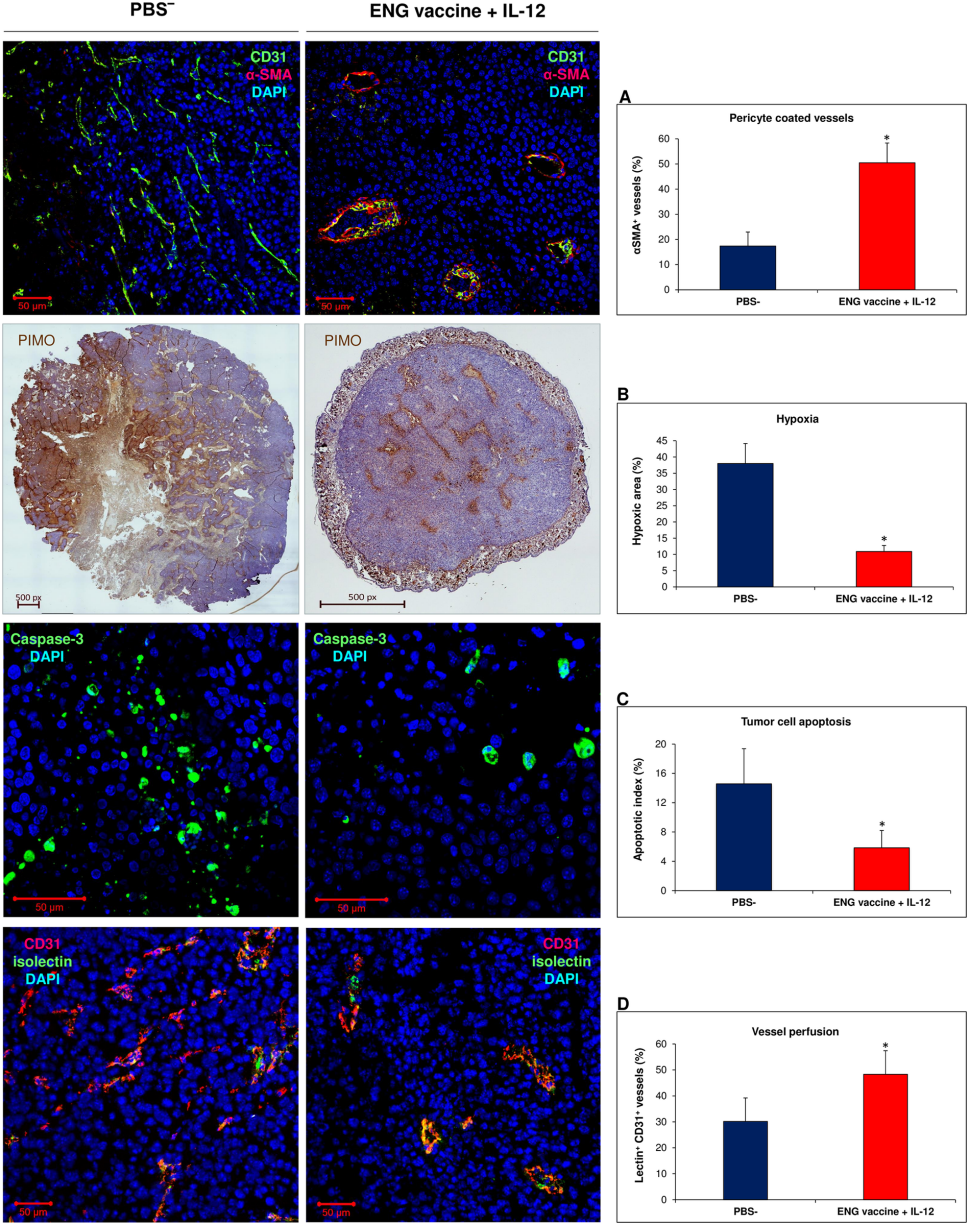
Effect of combined therapy (ENG vaccine + IL-12) on tumor blood vessels. On 20^th^ day of the combined therapy, mice were sacrificed and tumors were excised for immunohistochemical staining. “Normalized” vessels were identified using several tests [35]: (A) α-SMA and CD31 staining was used to identify pericytes-covered tumor vessels (α-SMA^+^CD31^+^ vessels, percentage of CD31^+^ vessels; n = 6; 10 visual fields per tumor section; magnification 20×; *P <0.001, the Cochran’s C test). (B) Staining with pimonidazole (PIMO) was conducted to visualize hypoxic regions in tumors (PIMO^+^ area (% of tumor area); n = 5; magnification 4×; *P <0.001, the Student’s test). (C) Caspase-3 staining was used to identify cancer cells that undergo apoptosis during vasculature “normalization” (apoptotic index: caspase-3^+^/ total cells; n = 6; 10 visual fields per tumor section; magnification 40×; *P <0.001, the Mann-Whitney U test). (D) Lectin perfusion test was used to assess vessel permeability in tumors (lectin^+^CD31^+^ vessels (% of CD31^+^ vessels); n = 6; magnification 20×; *P <0.001, the U Manna-Whitneya test). The structure of tumor vessels in mice treated with combined therapy resembles a regular one: with open lumens, the walls are thick, with a fine layer of pericytes (αSMA) adjacent to their surface (A). Smaller areas of hypoxia (B) and lower number of cells undergoing apoptosis were also found in tumors of treated mice (C). Increased number of perfused blood vessels (lectin^+^CD31^+^) were observed in tumor sections from mice treated with combined therapy (D). This indicated that the B16-F10 tumor vasculature in ENG vaccine with IL-12- treated mice is mature and functional.

There are several reports that indicate the role of M1-like macrophages in the normalization of malignant blood vessels [35–37]. We investigated the effect of combined therapy, which induced macrophages repolarization towards M1-like phenotype, on the structure of the tumor blood vessels. On the 20^th^ day of the experiment mice were sacrificed and tumors were collected for immunohistochemical analyses. “Normalized” vessels were identified using several tests [35]: α-SMA and CD31 staining was used to identify pericytes-covered tumor vessels; pimonidazole (PIMO) staining was conducted to visualize hypoxic regions in tumors; caspase-3 staining was used to identify cancer cells that underwent apoptosis during vasculature “normalization”; FITC-conjugated lectin was used to study perfusion (Fig 9). In previous report we observed decreased number of blood vessels (CD31) in tumor sections from mice treated with combined therapy [42]. Here, we observed in PBS¯ group (control) numerous small vessels, with narrow lumen. In addition, the walls of blood vessels contained fewer pericytes adhering to their surface. Blood vessels in tumors after combined therapy showed a similar structure to normal ones. The walls of the vessels had a well-visible layer of pericytes adhering to their surface (more than three times greater proportion of pericytes in the structure of blood vessels, compared to controls). In cross-sections of tumors of treated mice there were fewer blood vessels larger in size but characterized with a broader lumen compared to controls (Fig 9A). In addition, a 4-times larger area of hypoxia was observed in sections obtained from tumors of mice which received PBS¯ compared to tumors of mice treated with combined therapy (Fig 9B). Across the area of control tumors, nearly 15% of all cells were apoptotic. While in sections of tumors subjected to therapy, only 5% of the cells was recognized as apoptotic (Fig 9C). Following FITC-isolectin administration, we observed more than 1,5-time higher number of perfused blood vessels in tumors subjected to treatment compared to mice which received PBS¯ (Fig 9D).

**Fig 10:**
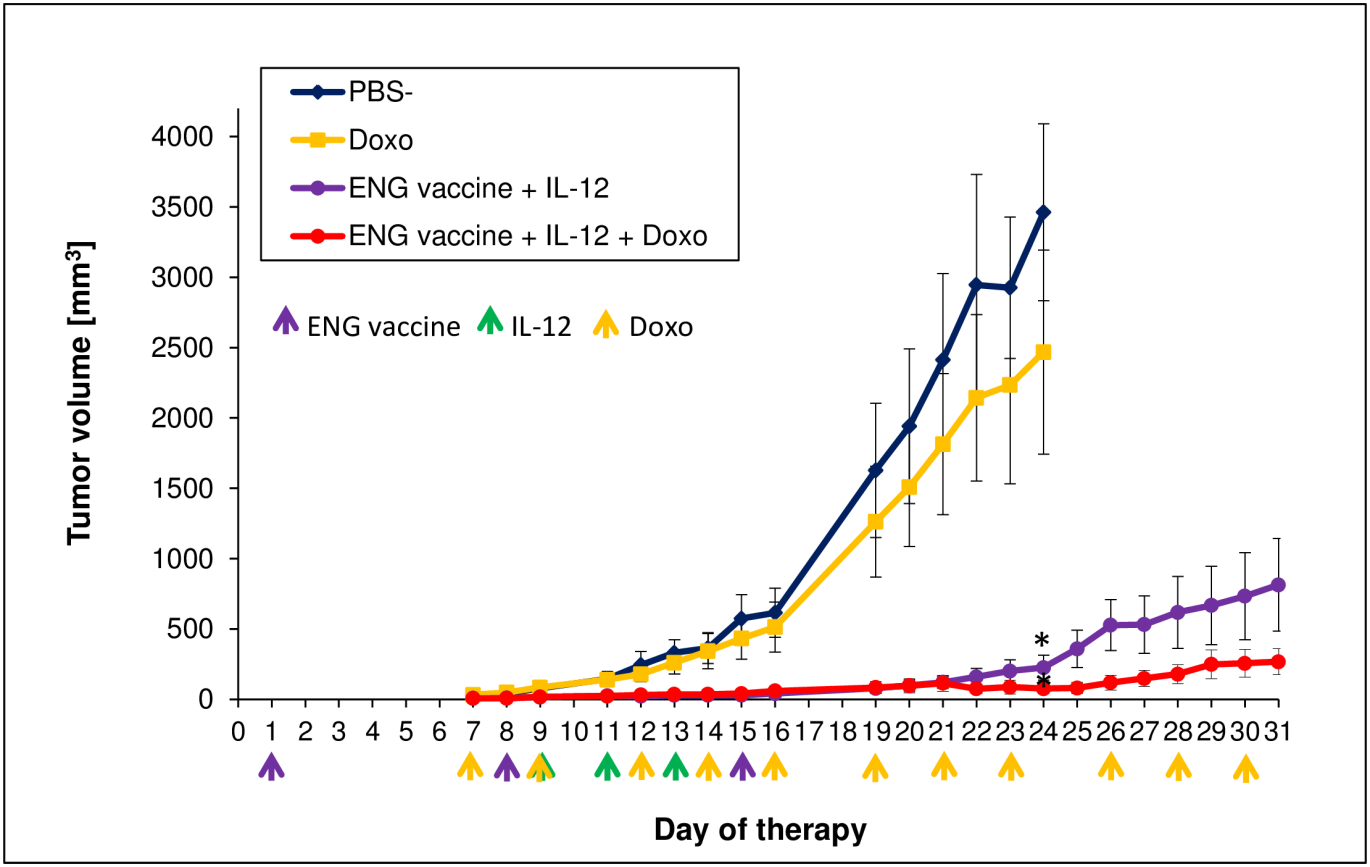
Inhibition of B16-F10 tumor growth in response to combination therapy involving endoglin-based DNA vaccine, IL-12 and chemotherapy. One day after inoculating mice (n = 6–7) with B16-F10 cells, the animals were orally vaccinated (three times at one-week intervals). Additionally, on days 9, 11 and 13 following inoculation with cancer cells, IL-12 was injected directly into tumors (Materials and methods). Moreover, doxorubicin (Doxo) was delivered intraperitoneally at a dose of 2.5 mg/kg, 3 times/week. “Control” mice received PBS¯ only. The suboptimal doses of the cytotoxic agent doxorubicin inhibited the growth of tumors in mice treated with combined therapy, but only slightly inhibited the tumor growth in controls. * *P*<0.01, the ANOVA followed by the Tukey’s *post hoc* test.

We then investigated whether improved vascular perfusion and, consequently, increased oxygenation in tumors increased the effectiveness of chemotherapy. For this purpose, mice treated with combined therapy (ENG vaccine + IL-12) were additionally treated with low doses of doxorubicin (2.5 mg/kg body mass, 3 times/week). We observed that suboptimal doses of doxorubicin increased the therapeutic effect of IL-12 vaccine and additionally inhibited the growth of tumors treated with the combination of drugs (ENG vaccine + IL-12). In the control group of mice receiving only PBS¯, suboptimal doses of doxorubicin slightly inhibited the growth of tumors (Fig 10). These data indicate that “normalized” blood vessels contributed to increased sensitivity of cancer cells to chemotherapy.

**Fig 11:**
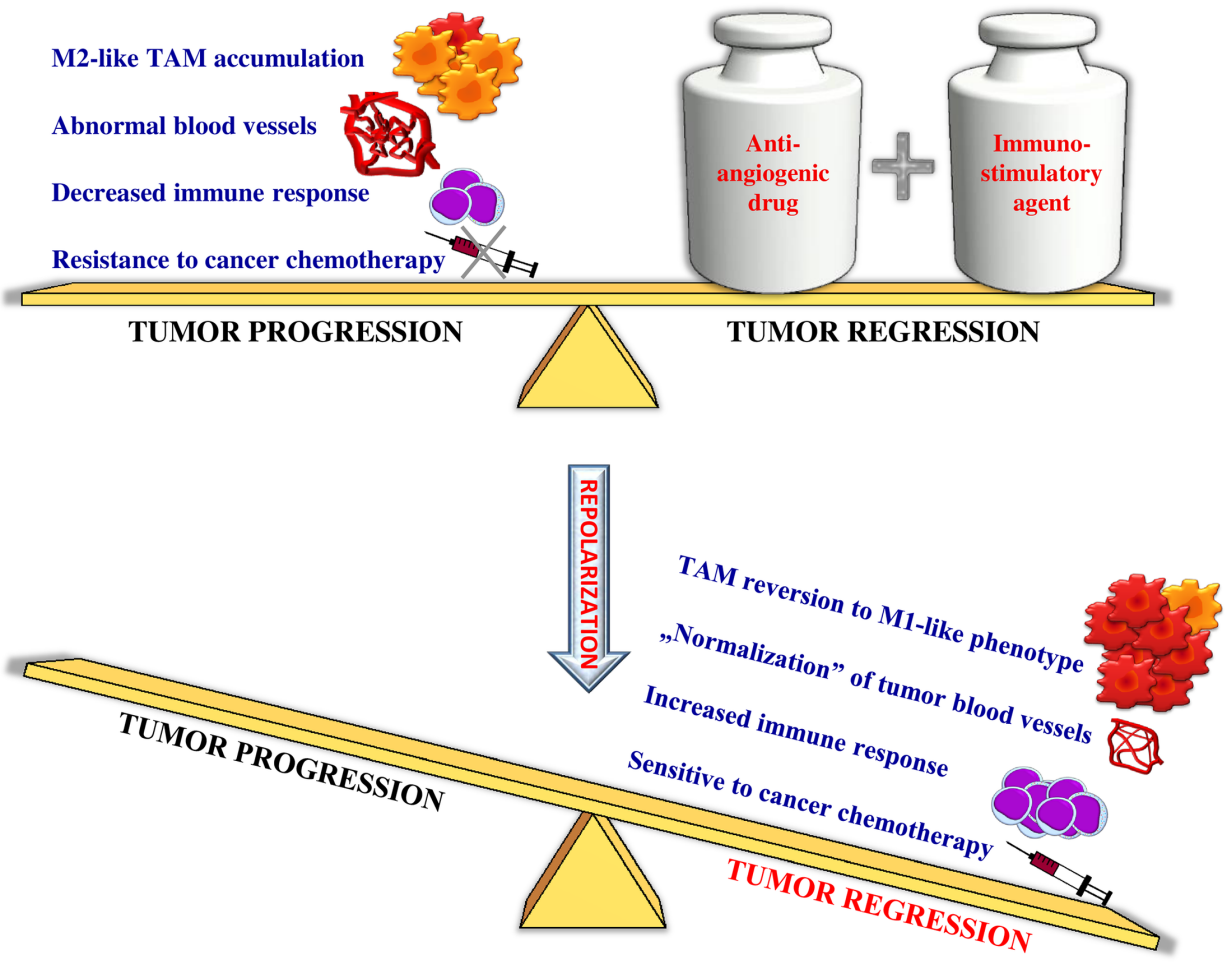
Repolarization of tumor-associated macrophages by endoglin-based DNA vaccine and IL-12. Progression of tumor strongly depends on the tumor microenvironment [1–7]. Cells that form tumor milieu, especially tumor-associated macrophages (TAMs), play a significant role in at least two key processes underlying neoplastic progression: angiogenesis and immune surveillance [5,9,10]. The structure of tumor blood vessels is defective and they are functionally abnormal [6,15–19]. Slowed-down circulation of blood leads to underoxygenation. Hypoxia stimulates formation of novel microvessels and invasiveness of tumor cells [6,20]. TAMs phenotypic changes play an important role in tumor vessel abnormalization/ normalization. M2-like macrophages stimulate immunosuppression and formation of defective tumor blood vessels and tumor progression. In contrast M1-like macrophages can activate immune response and cause normalization of irregular tumor vascular network which should sensitize cancer cells to chemo- and radiotherapy and lead to tumor growth regression [29,35–38]. Combination of antiangiogenic drug and immunostimulatory agent like the endoglin-based DNA vaccine with IL-12 repolarizes TAMs phenotype from M2-like (tumor growth-promoting) into M1-like (tumor growth-inhibiting) which affects the structure of tumor blood vessels and tumor regression.

Our results show that the combination of endoglin-based DNA vaccine with IL-12 repolarizes TAMs phenotype from M2-like (tumor growth-promoting) into M1-like (tumor growth-inhibiting) which affects the structure of tumor blood vessels and tumor regression (Fig 11).

## Discussion

Melanoma is one of the most aggressive type of skin cancer. Patients diagnosed with metastatic melanoma on average will not survive for more than 5 years. Despite recent advances in chemotherapy and immunotherapy, the available drugs are relatively toxic and act only on selected lesions [59]. In 2016, 10130 deaths due to melanoma were noted in USA, accounting for approximately 1.7% of all cancer-related deaths [60]. Between 2011 and 2014, FDA approved 7 new drugs designed for metastatic melanoma. These include monoclonal antibodies: Vemurafenib, Dabrafenib, Trametinib, Ipilimumab (anti-CTLA4), Pembrolizumab (anti-PD-1) and Nivolumab (anti-PD-1), and anti-proliferative cytokine used as adjuvant: Peginterferon alfa-2b. Cancer immunotherapy is a promising and specific therapeutic strategy for melanoma treatment and may involve passive antibody transfer, adoptive transfer of T cells and therapeutic vaccines [61].

The effectiveness of immunotherapy depends on the presence of immune cells in the tumor microenvironment. These cells play dual roles: the immune response can prevent and inhibit tumor growth, and immune cells in the tumor microenvironment may interact with tumor cells to promote tumor growth [4,10]. The cells of immune system present in tumors are: monocytes, macrophages, neutrophils, T and B lymphocytes, dendritic cells, immunosuppressive T_reg_ cells and myeloid-derived suppressor cells [2,4]. During the cancer progression the immune cells along with fibroblasts, myofibroblasts, mesenchymal stromal cells, endothelial cells and pericytes participate in immunosuppression and formation of tumor vascular networks. Cells in tumor milieu release proangiogenic agents which also act as immunosuppression stimulants [1,7–10].

During the anti-cancer treatment, which include anti-angiogenic drugs, the phenomenon of resistance is observed [4,14,62]. Our group has proposed a solution that avoids a number of resistance mechanisms [42]. The strategy includes a vaccine against endoglin in combination with interleukin 12. Endoglin (ENG) is overexpressed on the surface of endothelial cells but also on some cancer cells (among others murine B16-F10 melanoma cells) [40–44]. Endoglin mediates vascular maturation during angiogenesis [46]. The carrier of ENG therapeutic gene was an attenuated *Salmonella* Typhimurium SL7207 strain. The designed vaccine has many advantages: it is delivered orally; activates both specific and nonspecific immune response; induces immune response both against tumor blood vessels and tumor cells expressing endoglin on their surface; inhibits angiogenesis; inhibits the growth of primary tumors and metastases; does not affect the rate of wound healing [42]. IL-12 was administered directly into the tumor as an immunostimulatory factor [47–50] in a form of plasmid DNA. Combination of vaccine and IL-12 yields better therapeutic effects than either agent alone (30% of completely cured mice). We observed that combination of ENG vaccine with IL-12 lowers the level of regulatory T lymphocytes and the number of tumor blood vessels [42].

In this study, we examined repolarization of TAMs from M2- to M1-like phenotype in B16-F10 murine melanoma exerted by a combination of endoglin-based DNA vaccine and IL-12 and the effect of this reversion on tumor blood vessels. The therapeutic response of melanoma patients to immunotherapy is correlated with the presence of T lymphocytes in the tumor microenvironment. Whereas the impact of other cellular components of the microenvironment, including macrophages or mast cells, is underperformed [63]. We have focused on TAMs due to fact that depending on the macrophages phenotype they can either inhibit tumor growth (M1-like cells) or stimulate it (M2-like cells) [24,30–34]. “Classically-activated" M1 (“killing” phenotype) macrophages produce pro-inflammatory cytokines, participate in antigen presentation and play an antitumor role. In contrast, "alternatively-activated" M2 (“healing” phenotype) macrophages produce anti-inflammatory cytokines and stimulate tumor growth [3,22,24,32,63,64]. Furthermore most of TAMs exhibit an M2-like (proangiogenic) phenotype that promotes endothelial cell proliferation and tumor angiogenesis [22,24,37]. In addition, TAMs (M2-like) increase the tumor cells motility, invasiveness and immunosuppression [21,24,26]. Pro-tumor M2 macrophages inhibit the cytotoxic activity of the immune cells through the secretion of immunosuppressive factors. In contrast, M1 macrophages secrete proinflammatory cytokines that activate and recruit immune cells with anti-tumor properties such as cytotoxic T cells and NK cells, what retards tumor growth [65]. Our results demonstrate that combination of ENG-based DNA vaccine with IL-12 significantly increases the percentage of the tumor-infiltrating M1-like macrophages and reduces the percentage of the tumor-infiltrating M2-like macrophages (Fig 2). In melanoma, the density of TAMs correlates with the invasiveness of tumor cells and the poor prognosis [21,64,66]. M2-like macrophages were located mainly on the periphery of the tumor and in necrotic areas both in tumors obtained from treated and control groups of mice (Fig 2). Macrophages are attracted to hypoxic regions of tumor by hypoxia-induced chemoattractants secrete by tumor cells, i.e.: VEGF, endothelin, CCL2 [25,37]. The hypoxic environment modulates expression of TAMs genes towards M2 pro-tumor phenotype [10,67]. TAMs accumulation in hypoxic regions correlates with increased angiogenesis and invasive phenotype of tumor cells. The result of response to hypoxia in a growing tumor is a switch in macrophage polarization [3,27].

Furthermore, we observed that the ratio of M1-like (anti-tumor) TAMs to M2-like (pro-tumor) TAMs was more than three times increased (Fig 3). We observed increased expression of M1-type proinflammatory genes (*iNOS*, *IL-1b*, *CXCL-9*, *IL-12a*) and decreased expression of M2-type anti-inflammatory (*Arg-1*, *CCL-22*, *MRC-1*, *CCL-17*, *CSF-1)* and proangiogenic (*MMP-9*, *VEGFa*, *PlGF*, *VEGFc*) genes in TAMs from treated mice compared to controls. Only the level of expression *CXCL-11* gene, which is characteristic for M1 macrophages, was slightly reduced in tumors obtained from treated mice. In contrast, *IL-10* expression level, characteristic for M2 macrophages [36,64], was 4-times higher in tumors (with M1-like macrophages) from treated mice compared to controls (Fig 4). Interleukin 10 is considered to be immunosuppressive, anti-inflammatory cytokine produced by macrophages, regulatory T lymphocytes and epithelial cells [68–70]. Kubota et al. [71] observed robust production of IL-10 by TAMs, which influenced the invasiveness and metastasis of tumors. However, Wilke et al. [72] described the dual role of IL-10 in anti-cancer response and immunoregulation. Mumm et al. [68] demonstrated that IL-10 induces anti-cancer immunological surveillance mechanisms. IL-10 induces infiltration and activation of CD8^+^ tumor-specific lymphocytes, expression of Th1: IFN-γ cytokines and granzymes, and increases antigen presentation [68,70,73]. Our results indicate that M1-like macrophages present in the tumors of mice treated with combination therapy, which exhibit an enhanced expression of *IL-10*, also expressed reduced levels of anti-inflammatory (*Arg-1*, *CCL-22*, *CCL-17*, *CSF-1)* and proangiogenic (*MMP-9*, *VEGFa*, *PlGF*, *VEGFc*) genes. CXCL9 acts as proinflammatory and antitumor cytokine because it is capable of activating and recruiting NK cells and CD8^+^ T cells [65]. Increased expression of *CXCL9* in M1-like TAMs promotes tumor infiltration by CD8^+^ T lymphocytes and NK cells [36]. Among the investigated proangiogenic genes, we also observed decreased expression of *PlGF*. Rolny et al. [35] demonstrated that inhibition of tumor growth and metastasis results from the induction of polarization of macrophages and normalization of vessels by downregulation of *PlGF*. In summary, our data demonstrates the shift of the phenotype of tumor-infiltrating cells towards the anti-tumor M1-like phenotype. Besides, similarly to Huang et al. [36] and Fridlender et al. [53], we have observed that depletion of TAMs diminished the growth of control tumors, where we observed mainly M2-like phenotype of TAMs. Depletion of M2-like macrophages, which are tumor-promoting, reduces tumor growth. Furthermore, in mice subjected to combined therapy, we observed enhanced tumor growth after TAMs depletion. In tumors of treated mice we observed mainly M1-like phenotype of TAMs. Depletion of M1-like macrophages, which are tumor-inhibiting, abrogates antitumor effect of combined therapy (Fig 5). These data indicate an important role of TAM macrophages in progression/regression of tumors.

Interactions between innate immune system (*inter alia* by macrophages) and an adaptive immune system (including T lymphocytes) are essential for the prevention of tumor progression [74]. TAMs are immunosuppressive and affect lymphocytes infiltration. They produce chemokines including CCL-17, CCL-22, which recruit T regulatory cells (T_reg_), Th2 cells, and inhibit Th1-mediated response [75]. While M1-like can enhance recruitment and activation of CD8^+^ lymphocytes and NK cells [65]. In treated mice in which macrophage repolarization occurred, we observed increased infiltration of immune cells. Level of CD4^+^ lymphocytes was increased more than three times, CD8^+^ lymphocytes more than eight times and NK cells more than three times, compared with control tumors (Fig 6). NK cells, besides direct cytolytic effect against tumor cells, may shape adaptive immune response toward antitumor Th1 profile [76–77]. These cellular mediators of the innate immune system modulate dendritic cell and cytotoxic T cell maturation by production of cytokines [30,78]. Macrophages M1-like affect NK cells via cell-to-cell interaction and soluble interactions. This leads to activation of cytotoxicity in NK cells [79]. While CD4^+^ lymphocytes are helper cells and CD8^+^ lymphocytes are effector cells of adaptive cellular immunity. Many immunotherapies are aimed at activating these cells to promote tumor cell destruction [30]. In previous report we showed an effective induction of cytotoxicity of cytotoxic T-lymphocytes directed against endothelial and cancer cells overexpressing endoglin [42]. Similar to others [54,80] we observed a diverse effect on tumor growth after CD4^+^, CD8^+^ lymphocytes, and NK cells depletion. Depletion of CD8^+^ lymphocytes and NK cells, but not CD4^+^ lymphocytes, reduced the effect of combined therapy (Fig 7). It indicates the importance of CD8^+^ lymphocytes and NK cells in the therapeutic effect of ENG vaccine combined with IL-12. While depletion of CD4^+^ or CD4^+^CD25^+^ lymphocytes *in vivo* increases secretion of IFN-ɣ by CD8^+^ lymphocytes and significantly reduces growth of tumors and enhances survival [80]. Like Wallerius et al. [65], by depletion of macrophages, we were able to show that tumor growth suppression by combination therapy is dependent on the phenotype of the macrophages inside the tumor, which in turn controls the recruitment of cytotoxic lymphocytes. Depletion of macrophages leads to reduced recruitment of CD8^+^ lymphocytes (Fig 8). M1-like macrophages induce T lymphocytes infiltration into tumors and increase their ability to kill tumor cells [65]. TAM macrophages suppress activation of CD8^+^ lymphocytes by several mechanisms: removal of metabolites essential for T-cell proliferation, T-cell suppression by the production of anti-inflammatory cytokines, and activation of T-cell checkpoint blockade by inhibitory receptors [74].

Rolny et al. [35] and Huang et al. [36] observed that antitumor therapy, which affects the phenotype of macrophages and skews TAMs polarization away from the M2- to a tumor-inhibiting M1-like phenotype, also influences malignant blood vessels. The process of tumor blood vascular network development considerably affects growth and progression of cancer cells [11–15]. Structure of tumor blood vessels is defective and they are functionally abnormal [6,15–19]. Slowed-down circulation of blood leads to underoxygenation (hypoxia) and necrosis of cells present in the vicinity of such blood vessels [6,20]. The use of appropriate doses of antiangiogenic agents can normalize tumor vasculature, reduce hypoxia, increase penetration of drugs and antitumor immune cells and thus increase the effectiveness of other therapies [9,15,18,67,81–84]. The process of angiogenesis and normalization of blood vessels is influenced by the dynamic changes of TAMs phenotype [35–37]. While M2-like cells participate in the formation of abnormal dysfunctional blood vessels, M1-like cells tend to “normalize” tumor blood vasculature [35–38]. We have observed a similar effect of our therapy on malignant blood vessels. The structure of tumor vessels in mice treated with combined therapy resembled a regular one: the walls were thick with an increased pericyte coverage. Higher number of perfused blood vessels, smaller areas of hypoxia and lower level of cancer cells undergoing apoptosis were also found in tumor sections obtained from treated mice (Fig 9). In solid tumors, hypoxic conditions often induce apoptosis of tumor cells. Apoptotic tumor cells may affect TAMs to upregulate the production of proangiogenic prostaglandin E2 (PGE2) [37]. Macrophages by participating in formation of abnormal and hypoperfusive vessels, that restrict the delivery of chemotherapeutic agents, induce resistance to chemotherapy. Depletion of TAMs increases the effectiveness of chemotherapy due to vessels normalization. It improves the drug delivery process by increasing tumor blood supply [37]. Like Rolny et al. [35] we used suboptimal doses of doxorubicin and observed inhibited growth of tumors in mice subjected to combined therapy, but only slightly inhibited growth of control tumors (Fig 10). Our data indicated that B16-F10 vasculature in ENG vaccine with IL-12- treated mice is mature. Functional and “normalized” blood vessels contributed to increased sensitivity of cancer cells to chemotherapy.

To conclude, when designing new therapeutic strategies, apart from targeting tumor cells, therapies that transform tumor microenvironment should be considered. Progression of tumor strongly depends on the tumor microenvironment. TAMs (M2-like) play a significant role in angiogenesis and immune surveillance. Reverting macrophages from M2-like phenotype towards M1-like phenotype is a promising anticancer therapeutic strategy which induces normalization of cancer blood vessels. Here, we showed that combination of antiangiogenic drug and immunostimulatory agent like the endoglin-based DNA vaccine with IL-12 can repolarize TAMs phenotype from M2-like (tumor growth-promoting) into M1-like (tumor growth-inhibiting) which affects the structure of tumor blood vessels, tumor infiltration by immune cells and tumor regression (Fig 11).
